# Biophysical features of MagA expression in mammalian cells: implications for MRI contrast

**DOI:** 10.3389/fmicb.2014.00029

**Published:** 2014-02-05

**Authors:** Anindita Sengupta, Karina Quiaoit, R. Terry Thompson, Frank S. Prato, Neil Gelman, Donna E. Goldhawk

**Affiliations:** ^1^Imaging Program, Lawson Health Research InstituteLondon, ON, Canada; ^2^Medical Biophysics, Western UniversityLondon, ON, Canada; ^3^Collaborative Graduate Program in Molecular Imaging, Western UniversityLondon, ON, Canada

**Keywords:** magnetic resonance imaging, MagA, modified ferritin subunits, relaxation rates, iron, cancer cells

## Abstract

We compared overexpression of the magnetotactic bacterial gene *MagA* with the modified mammalian ferritin genes *HF + LF*, in which both heavy and light subunits lack iron response elements. Whereas both expression systems have been proposed for use in non-invasive, magnetic resonance (MR) reporter gene expression, limited information is available regarding their relative potential for providing gene-based contrast. Measurements of MR relaxation rates in these expression systems are important for optimizing cell detection and specificity, for developing quantification methods, and for refinement of gene-based iron contrast using magnetosome associated genes. We measured the total transverse relaxation rate (R2*), its irreversible and reversible components (R2 and R2′, respectively) and the longitudinal relaxation rate (R1) in MDA-MB-435 tumor cells. Clonal lines overexpressing MagA and HF + LF were cultured in the presence and absence of iron supplementation, and mounted in a spherical phantom for relaxation mapping at 3 Tesla. In addition to MR measures, cellular changes in iron and zinc were evaluated by inductively coupled plasma mass spectrometry, in ATP by luciferase bioluminescence and in transferrin receptor by Western blot. Only transverse relaxation rates were significantly higher in iron-supplemented, MagA- and HF + LF-expressing cells compared to non-supplemented cells and the parental control. R2* provided the greatest absolute difference and R2′ showed the greatest relative difference, consistent with the notion that R2′ may be a more specific indicator of iron-based contrast than R2, as observed in brain tissue. Iron supplementation of MagA- and HF + LF-expressing cells increased the iron/zinc ratio approximately 20-fold, while transferrin receptor expression decreased approximately 10-fold. Level of ATP was similar across all cell types and culture conditions. These results highlight the potential of magnetotactic bacterial gene expression for improving MR contrast.

## INTRODUCTION

Medical imaging provides a non-invasive means of monitoring disease processes from diagnosis through therapy, and is an essential component of healthcare today. Among the available imaging platforms, magnetic resonance imaging (MRI) is preferred for many reasons, including superb anatomic detail, at any tissue depth (Burtea et al., 2008). Despite these strengths, MRI does not yet have the tools to effectively track cellular and molecular activities, as has been achieved in optical imaging using reporter genes such as the green fluorescent protein and luciferase. Development of molecular imaging methods to track mammalian cells using MRI requires refinement of both contrast gene expression systems and magnetic resonance (MR) detection methods.

Gene-based iron-labeling for MRI has broad interest owing to the paramagnetic and superparamagnetic properties of iron. Various iron handling proteins and mechanisms have been examined for their potential as MR contrast agents, including those long recognized for their key role in mammalian iron homeostasis: iron response elements (Genove et al., 2005), ferritin subunits (Cohen et al., 2009), and transferrin receptor (Deans et al., 2006). Part of the challenge in adapting iron binding proteins for use in generating MR contrast relates to the elaborate control of iron homeostasis in mammalian cells (Pantopoulos et al., 2012) and the manner in which this may fluctuate in response to changes in physiological state (Recalcati et al., 2010). An ideal method for generating iron nanoparticles for molecular MRI would be subject to molecular regulation of magnetite formation and compatible with cellular iron homeostasis.

Magnetotactic bacteria are an extraordinary example of how single cells may synthesize and compartmentalize an iron biomineral and harness its magnetic properties (Komeili, 2012). The functional unit is a magnetosome and typically consists of a lipid bilayer surrounding a magnetite/maghemite crystal. This subcellular structure is assembled in a protein-directed manner and may be largely encoded on a magnetosome genome island (Richter et al., 2007; Jogler and Schuler, 2009). While definition of the molecular nature of the magnetosome is steadily building, multiple applications of the magnetosome are being developed and refined, testifying to the utility of this unique prokaryotic compartment (Takahashi et al., 2009; Yan et al., 2012). A fuller understanding of which genes are essential for the synthesis of the basic magnetosome compartment and for the manipulation of select magnetosome features, would permit the versatile use of this structure in the generation of MR contrast for pre/clinical imaging (Goldhawk et al., 2012). Features such as the size and shape, composition, and clustering of the iron biomineral may provide distinct MR signals for the detection of cellular and molecular activities. In addition, the superparamagnetic property of magnetite provides a more effective MR contrast agent than, for example, the ferrihydrite core of ferritin.

We have used the putative iron transporter *MagA* (Nakamura et al., 1995b) as a prototype for magnetotactic bacterial and magnetosome gene expression in mammalian cells, to enhance MR contrast and, in the future, enable effective reporter gene expression for MRI. Although not an essential magnetosome gene (Uebe et al., 2012), we showed that overexpression of *MagA* from AMB-1 provides MR contrast comparable to the overexpression of modified, mammalian ferritin subunits (*HF + LF*) that are devoid of iron regulatory elements (Rohani et al., 2013). In a mouse model of tumor growth, transplanted cells were repetitively imaged over 5 weeks and compared to the parental cell xenograft. Both MagA- and HF + LF-expressing tumors provided contrast enhancement. Moreover, MagA-derived contrast exhibited greater contrast to noise ratio than HF + LF-expressing tumors, particularly in the immediate days post-injection, indicating a role for select magnetotactic bacterial genes in preclinical molecular imaging.

More precise localization of iron-loaded cells in the developing tumor may be derived from a quantitative measure of iron contrast such as relaxation rate mapping. The report herein investigates the longitudinal (R1) and transverse (R2*, R2, and R2′) relaxation rates in MagA- and HF + LF-expressing MDA-MB-435 cells. Using 3 Tesla (T) MRI and gelatin phantoms, we show the manner in which the cellular MR signal changes as a function of iron supplementation. Results are examined in light of elemental iron and zinc content, reflecting iron uptake and cellular redox status, as well as ATP and transferrin receptor levels, addressing the active transport and regulation of iron uptake. These findings highlight the utility of a single magnetotactic bacterial gene as an MR contrast agent, subject to genetic control. We predict the potential for further improvements in MR detection of gene-based contrast upon fuller delineation of the magnetosome compartment.

## MATERIALS AND METHODS

### CELLS

Human MDA-MB-435 breast/melanoma cells were stably transfected with *MagA* (Goldhawk et al., 2009) and *HF + LF* (Rohani et al., 2013) as previously described. Briefly, clonal cell lines were cultured in low glucose Dulbecco’s Modified Eagle Medium supplemented with 10% fetal bovine serum and 0.5% penicillin/streptomycin. Iron-supplemented cells were prepared by incubation with medium containing 250 μM ferric nitrate (Sigma-Aldrich, Oakville, ON, Canada) for at least 5 days. All cell culture reagents were purchased from Life Technologies (Burlington, ON, Canada) unless otherwise noted.

**FIGURE 1:**
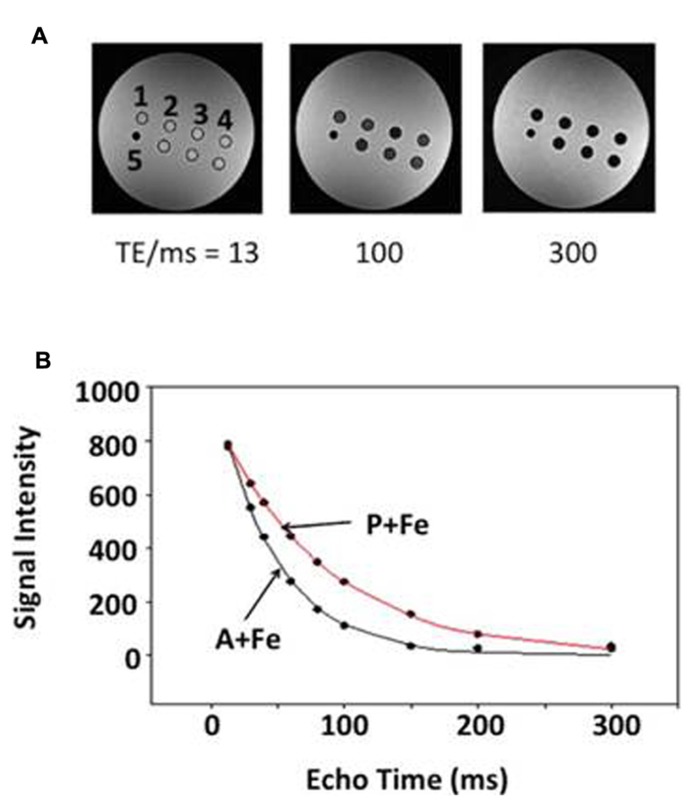
**Relaxation rate measurement in a spherical phantom.** Representative data indicate the influence of echo time (TE) on signal decay. **(A)** Single-echo SE images show sample wells in cross section at three different TE values (13, 100, and 300 ms): 1, parental (P); 2, MagA (A); 3, iron-supplemented MagA (A + Fe); 4, iron-supplemented parental (P + Fe); and 5 polystyrene marker for reference. Samples along the bottom row are combinations of MagA-expressing and parental cells. **(B)** R2 relaxation curves are shown for iron-supplemented samples. Symbols indicate the mean signal intensity within a region of interest (ROI) at each TE. Curves represent the best fit to an exponential decay.

Cultures were grown to confluency on 150 mm dishes; harvested by trituration; and washed three times with phosphate buffered saline pH 7.4 (PBS) to remove extracellular iron, centrifuging 5 min at 400 × *g* and 15°C. Cells were counted using a hemacytometer and 30 million cells of each type were placed in 1% gelatin/PBS in the wells from a 96-well break-apart plate (Nunc, Rochester, NY, USA). Each well was centrifuged to form a compact pellet 6 mm in height. Cell pellets were overlaid with 1% gelatin/PBS and embedded in one hemisphere of a 9 cm spherical phantom filled with 4% gelatin/PBS (**Figure 1A**). Samples consisted of either parental, MagA- or HF + LF–expressing cells, cultured in the presence and absence of iron supplementation. To form the spherical gelatin phantom, the empty hemisphere was filled with 2% gelatin/PBS and placed on top of the half containing cell samples. To avoid susceptibility artifacts at the interface, air was excluded using a layer of parafilm. In order to minimize macroscopic magnetic field inhomogeneities which would interfere with accurate R2′ measurement, we used a spherical-shaped phantom.

### RELAXATION RATE MAPPING

To quantify the MRI signal changes associated with cellular iron uptake, phantoms were scanned on a 3T Biograph mMR (Siemens AG, Erlangen, Germany) equipped with an actively shielded whole-body gradient system (45mT/m @ 200T/m/s). A 15-channel knee coil was used for radiofrequency (RF) excitation and signal reception. Quantification is based on measurement of the MRI relaxation rates. For R1 measurements, an inversion-recovery spin-echo (SE) sequence was used [TR (repetition time) = 4000 ms; TE (echo time) = 13 ms; FOV (field of view) = 120 mm; matrix = 128 × 128; slice thickness = 1.5 mm; flip angle = 90°], with inversion times of 22, 200, 500, 1000, 2000, and 3900 ms. For R2 measurements, a single-echo SE sequence was used with varying echo times (TR = 1000 ms; 9 echoes; TE = 13, 20, 30, 40, 60, 100, 150, 200, and 300 ms; FOV = 120 mm; matrix = 192 × 192; slice thickness = 1.5 mm; flip angle = 90°). Finally, R2* was measured with a multi-echo gradient echo sequence (TR = 1000 ms; 12 echoes; TE = 4.7, 11, 17, 23, 30, 36, 42, 49, 55, 61, 68, and 80 ms; FOV = 120 mm; matrix = 192 × 192; slice thickness = 1.5 mm; flip angle = 60°). Voxel dimensions were 1.5 × 0.6 × 0.6 mm for R2 and R2* acquisitions and 1.5 × 0.9 × 0.9 mm for R1 acquisitions. Image processing and analysis were performed using MATLAB 7.9.0 (R2010b), Excel 2010 (Version 14) and Sigma Plot 10.0.Ink. The region of interest (ROI) was selected in each well to include the maximum number of voxels (approx. 30–50), excluding ones adjacent to the wall of the well. R2*, R2, and R1 were determined with least square curve fitting of the mean ROI signals using standard equations. R2′ was obtained by subtraction (R2*–R2).

### ICP–AES/MS

Trace element analysis of iron and zinc was performed by the Analytical Services Laboratory of Western University (London, ON, Canada) using inductively coupled plasma atomic emission spectroscopy (ICP–AES) or mass spectrometry (ICP–MS). Zinc provided a measure of cellular redox status as well as a point of comparison to iron content. Cultured cells were lysed in RIPA/protease inhibitors as described below, such that approximately 10 million cells were solubilized per ml of lysis solution. Iron and zinc content were normalized to quantity of protein as determined by the BCA assay (Smith et al., 1985).

### ATP ASSAY

To assure cellular viability and capacity for active transport of iron, ATP content was measured following published procedures, with an ATP Bioluminescent Assay kit (Sigma-Aldrich, St. Louis, MO, USA) and a modified spectroscopy system (PTI, London, ON, Canada; Belton et al., 2008). Immediately following MR scanning, 50–100 μL cells were extracted from the gelatin phantom; mixed with 8 M Guanidine-HCl/10 mM EDTA at a ratio of 1 part cells: 9 parts lysis solution; and stored at -20°C for up to 12 h (Linklater et al., 1985). The ATP assay reaction was placed in a glass tube (Fisher Scientific, Nepean, ON, Canada) and consisted of 30 μL cell lysate, 570 μL sterile water, 600 μL 25 mM HEPES pH 7.75, and 500 μL dilution buffer provided with the kit. Immediately prior to measuring bioluminescence, 100 μL luciferin/luciferase solution was added to the sample and mixed for 10 s using a vortex. Bioluminescence was recorded for at least 2 min using Felix32 software (PTI, London, ON, Canada) to establish the peak value. ATP content was determined from an external ATP standard curve. Viability was assessed relative to control samples prepared from cultured cells, harvested and frozen at -80°C as cell pellets prior to the addition of lysis solution. ATP content was normalized to quantity of protein as described above.

### WESTERN BLOT

Sodium dodecyl sulfate polyacrylamide gel electrophoresis (SDS-PAGE; Laemmli, 1970) and Western blotting (Tobin et al., 1979) were performed according to published protocols with the following modifications. Protein samples were resolved on a 7% mini gel and transferred to a nitrocellulose membrane for 7 min using the iBlot Gel Transfer Device (Life Technologies). The membrane was blocked in 3% bovine serum albumin/10 mM Tris-HCl pH 7.4 buffered saline (TBS) for 3 h; incubated 18 h in a 1/1000 dilution of monoclonal rabbit anti-human transferrin receptor (α-TfR, EPR4012, Novus Biologicals, Oakville, ON, Canada)/TBS/0.02% sodium azide; followed by 2 h in a 1/5000 dilution of horse radish peroxidase (HRP) conjugated-goat anti-rabbit immunoglobulin (Santa Cruz Biotechnology, Dallas, TX, USA). All incubations were performed at room temperature and blots were washed with TBS/0.1% Tween 20. Immunoreactive bands were detected using an enhanced chemiluminescent HRP substrate (SuperSignal West Pico, Thermo Scientific, Rockford, IL, USA) and captured using GeneSnap software, version 7.09, and a Chemi Genius2 Bio Imaging System (SynGene, Cambridge, England). Densitometry was performed using GeneTools software, version 3.06.04 (SynGene).

Samples were prepared from cultured cells on 100 mm dishes, washed twice with PBS prior to harvesting in 1 mL RIPA (10 mM Tris-HCl pH 7.5/140 mM NaCl/1% NP-40/1% sodium deoxycholate/0.1% SDS)/150 μL Complete Mini protease inhibitor cocktail (Roche Diagnostic Systems, Laval, QC, Canada). Cell lysates were quantified and 50 μg of denatured protein was loaded into each lane. Molecular size was approximated using Novex Sharp Pre-stained Protein Standards (Life Technologies).

### STATISTICS

Relaxation rate means, standard deviations (SDs) and standard errors of the mean (SEM) were calculated for all group values. Assuming that the data were non-parametric, for each cell type (parental, MagA, and HF + LF), group mean relaxation rates (R2*, R2, and R2′) were compared between runs, with versus without iron supplementation, using the non-parametric Kruskal–Wallis test. The same non-parametric test was then used to compare all runs with iron versus all runs without iron, combining data from all three cell types. Finally, four linear regression models were tested with each relaxation rate measure as the dependent variable, and cell type and iron status (added/not added) as independent. To adjust for multiple comparisons, *p* = 0.01 was set as the threshold for statistical significance, with *p* = 0.10 as the threshold for statistical trend. The decision to adopt an intermediately conservative p value, rather than the most conservative Bonferroni adjusted threshold of *p* = 0.05/10 = 0.005, takes into consideration the relatively small sample size. All tests were two-tailed and SPSS version 21.0 was the statistical package used. Variation in ATP content among cell types and culture conditions was also evaluated using the Kruskal–Wallis test and *p* = 0.01, again adjusting for multiple comparisons.

## RESULTS

### RELAXATION RATES AND IRON CONTENT

Parental, MagA- and HF + LF-expressing cells were cultured in the presence and absence of iron supplementation and mounted in a spherical phantom to determine both longitudinal and transverse relaxation rates. **Figure 1A** illustrates the phantom set up for parental and MagA-expressing cells and provides representative data from R2 measurements. **Figure 1B** indicates the rate of signal decay with respect to TE. The manner in which contrast gene expression alters MR relaxation rates of cells cultured under iron-supplemented conditions is described further below.

**FIGURE 2:**
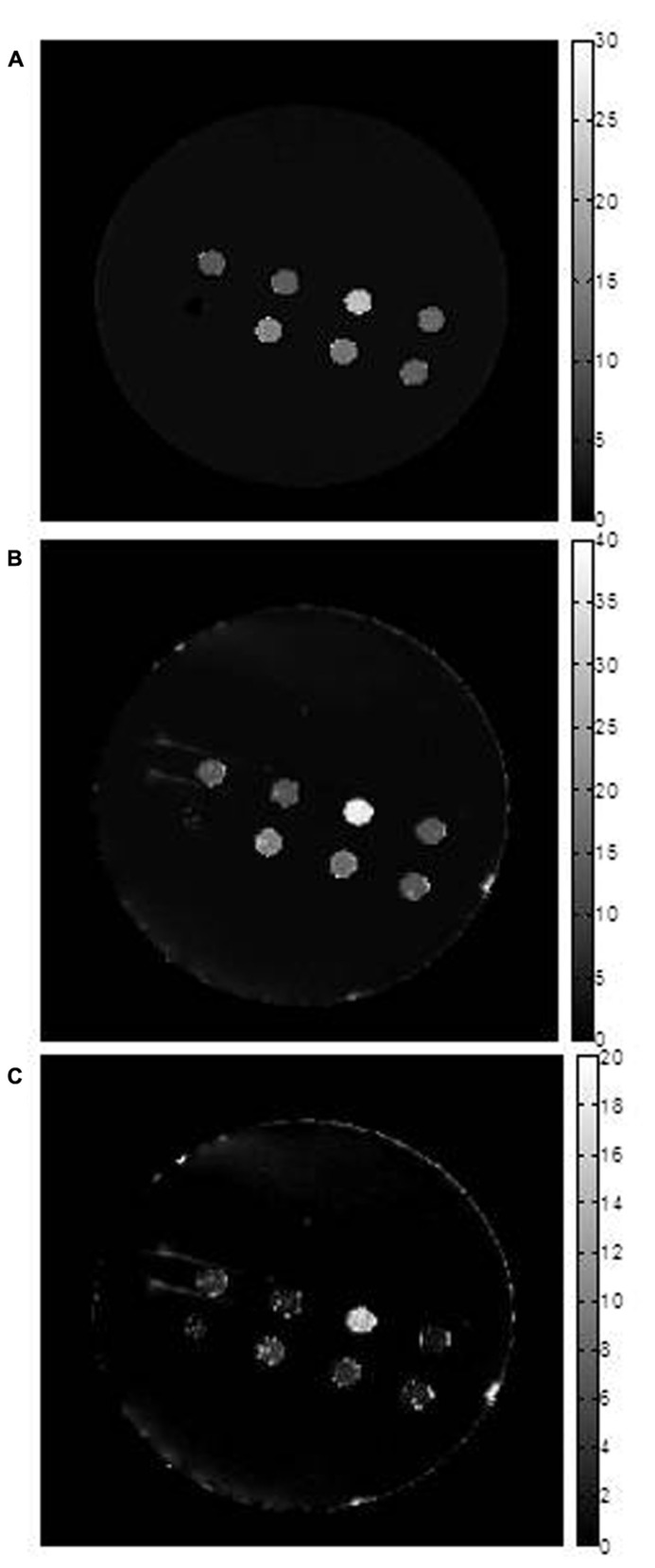
**Transverse relaxation rate mapping.** Representative maps are shown for **(A)** R2, **(B)** R2^*^ and **(C)** R2’. The first two maps were obtained using voxel by voxel curve fitting with an exponential decay function and the R2’ map was obtained by subtraction (R2^*^–R2). The units of the scale bar are sec^-1^. Images show sample wells in the phantom, in cross section. From left to right across the top row are: parental (P); MagA (A); iron-supplemented MagA (A + Fe); and iron-supplemented parental (P + Fe). Along the bottom row, from left to right, there is a polystyrene marker for reference and samples with combinations of iron-supplemented, MagA-expressing and parental cells. These samples decrease from 70% A + Fe to 50% and 30% and were not further evaluated. Note that pixel values for all three rates are highest for A + Fe. Maps are provided for display only; relaxation rates (**Table 2**) were determined as outlined in methods.

In all cell types and culture conditions, R1 remained virtually constant between 0.72–0.91 s^-^^1^ (**Table 1**) and was not pursued as an indicator of cellular iron contrast. On the other hand, transverse relaxation rates were notably different in iron-supplemented cells overexpressing MagA and HF + LF (**Figure 2**). Comparison of the total transverse relaxation rate, R2*, and its irreversible and reversible components, R2 and R2′ respectively, showed no significant differences in MR contrast of parental cells cultured in the presence or absence of iron supplementation (**Table 2**). However, the same non-parametric bivariate analysis of MagA-expressing cells exhibited a statistically significant influence of iron supplementation on all transverse relaxation rates. A similar response to iron supplementation was obtained in both MagA- and HF + LF-expressing cells. Overall, R2* provided the greatest absolute difference in MR contrast from iron-supplemented MagA- and HF + LF-expressing cells, while R2′ displayed the greatest relative difference.

**Table 1:** Longitudinal relaxation rates in parental, MagA- and HF + LF-expressing cells +/- iron supplementation.

Sample^a^	R1 (s^–1^)^b^	*n*
P	0.719, 0.821	2
P + Fe	0.762, 0.767	2
A	0.808 ± 0.025	5
A + Fe	0.812 ± 0.030	5
F	0.906 ± 0.053	3
F + Fe	0.794 ± 0.018	3

a Cells were incubated in the presence (+Fe) or absence of 250 μM ferric nitrate. P, parental; A, MagA expression; F, HF + LF expression.

b Individual values are reported for *n* = 2. Mean + SEM is reported for *n* = 3–5.

**Table 2:** Transverse relaxation rates in parental, MagA- and HF + LF-expressing cells +/**–** iron supplementation (non-parametric bivariate analysis).

Relaxation rate^^^ (s^–1^)	Parental (*n* = 4)			MagA (*n* = 8)			HF + LF (*n* = 5)		
	Fe (–) (SD)	Fe (+) (SD)	*p*	Fe (–) (SD)	Fe (+) (SD)	*p*	Fe (–) (SD)	Fe (+) (SD)	*p*
R2*	13.70 (3.07)	15.68 (5.83)	0.66	13.91 (3.69)	26.46 (8.69)	< 0.005	13.09 (1.87)	28.39 (11.59)	[< 0.05]^§^
R2	9.69 (0.76)	11.58 (2.30)	0.15	11.17 (2.95)	17.37 (4.80)	< 0.01	9.46 (2.31)	16.63 (6.35)	[< 0.05]
R2′	4.01 (2.41)	4.10 (3.94)	0.56	2.74 (1.03)	9.09 (4.12)	0.001	3.63 (2.04)	17.87 (20.66)	0.18

^ Mean +/**-** SD

§ Data in brackets represent a statistical trend, as identified by 0.01 < *p* < 0.10.

Across the entire sample, the significant effect of iron supplementation is confirmed using both bivariate and multi-variate analyses (**Table 3**). Kruskal–Wallis analysis indicates there is no significant influence of the expression system itself. Rather, it is the addition of an iron-supplement that exploits the difference in transverse relaxation rates in engineered cells. This finding is supported by linear regression analysis, which further indicates that the cell type variable approaches a statistically significant influence on R2′, the most iron-specific measure.

**Table 3:** Statistical analysis of transverse relaxation rates in parental, MagA- and HF + LF-expressing cells +**/** - iron supplementation.

Condition (*n* = 34)^a^	Kruskal–Wallis (bivariate)		
	R2*	R2	R2′
Iron vs. no iron	*p* = 0.001	*p* < 0.001	*p* < 0.005
P vs. A vs. F	*p* = 0.32	*p* = 0.24	*p* = 0.53

Condition (*n* = 34)^a^	Linear regression (multi-variate)		
	R2*	R2	R2′

Iron vs. no iron	*p* < 0.005	*p* < 0.001	(*p* < 0.05)^b^
P vs. A vs. F	*p* = 0.11	*p* = 0.27	(*p* = 0.09)

a Sample size, n, consists of 8 parental (P), 16 MagA (A) and 10 HF + LF (F). Data sets include both iron-supplemented and unsupplemented cells.

b Data in parentheses represent a statistical trend, as identified by 0.01 < *p* < 0.10.

Over the course of relaxation rate measurements, iron content was periodically evaluated in cultured cells. In any cell type cultured in the absence of iron supplementation, ICP–MS analysis indicated 0.048 ± 0.017 μg Fe/mg protein (*n* = 10; mean +/- SD) and 0.223 ± 0.121 μg Zn/mg protein (**Table 4** and data not shown). In contrast, the presence of iron supplementation in MagA- and HF + LF-expressing cells increased iron content to approximately 0.66 μg/mg protein while zinc levels remained at approximately 0.13 μg/mg protein (**Table 4**). The ratio of iron/zinc in non-supplemented cultures (approx. 0.22) is over 20 times lower than under iron-supplemented conditions (approx. 5.2). Interestingly, sizable increases in iron content were observed periodically in supplemented cultures of both MagA- and HF + LF-expressing cells, giving greater than 1 μg Fe/mg protein (data not shown since these samples exceeded the upper calibration range of ICP–MS, 0.172 + 0.012 μg Zn/mg protein, Fe/Zn over 6, *n* = 3, mean +/- SD).

**Table 4:** Trace element analysis of cells cultured in the presence or absence of iron supplementation^*^.

Sample	Iron^#^	Zinc^#^	Fe/Zn	*n*
A	0.047 ± 0.006	0.249 ± 0.047	0.19	7
A + Fe	0.667 ± 0.111•	0.117 ± 0.008_°_	5.70	7
F	0.044 ± 0.008	0.183 ± 0.034	0.24	5
F + Fe	0.650 ± 0.113•	0.139 ± 0.015_°_	4.68	4

*Data were collected using either ICP–AES or –MS.*

* Cells were incubated in the presence (+Fe) or absence of 250 μM ferric nitrate.

# Elemental analysis is reported as μg/mg protein; mean ± SEM.

• Samples +/- Fe were evaluated using the Student’s *t*-test and showed significance at *p* < 0.01.

° Samples +/- Fe were evaluated using the Student’s *t*-test and were not significant: *p* > 0.01.

### ATP STORES AND TRANSFERRIN RECEPTOR LEVELS

**FIGURE 3:**
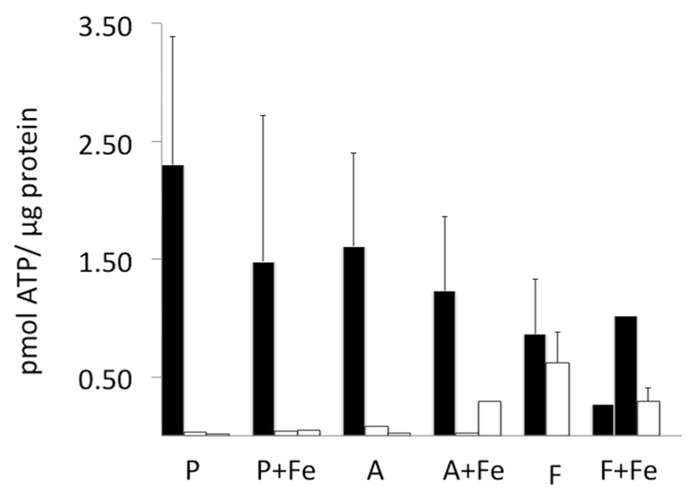
**Cellular ATP content before and after MR scanning.** ATP was quantified in each cell type using a luciferase bioluminescence assay and normalized to protein content (black bars). In parental and MagA-expressing cells, ATP content was not statistically different in the presence and absence of iron supplementation. In cells mounted in a gelatin phantom (open bars), the ATP content decreased variably within 24 h of harvest and scanning. Error bars represent SEM where *n* = 3–4; both values are shown where *n* = 2.

To address the potential for variation in amount of iron uptake in MagA- and HF + LF-expressing cells, we examined cellular levels of ATP and transferrin receptor. The ATP content of viable and scanned cell samples was evaluated using a luciferase bioluminescence assay and normalized to total protein (**Figure 3**). In general, viable cells contained approximately 0.50–2.50 pmol ATP/μg protein. There was no significant influence of iron supplementation or cell type on ATP levels. As expected, samples harvested within 24 h of scanning showed decreases in ATP content to levels generally less than 0.50 pmol ATP/μg protein.

**FIGURE 4:**
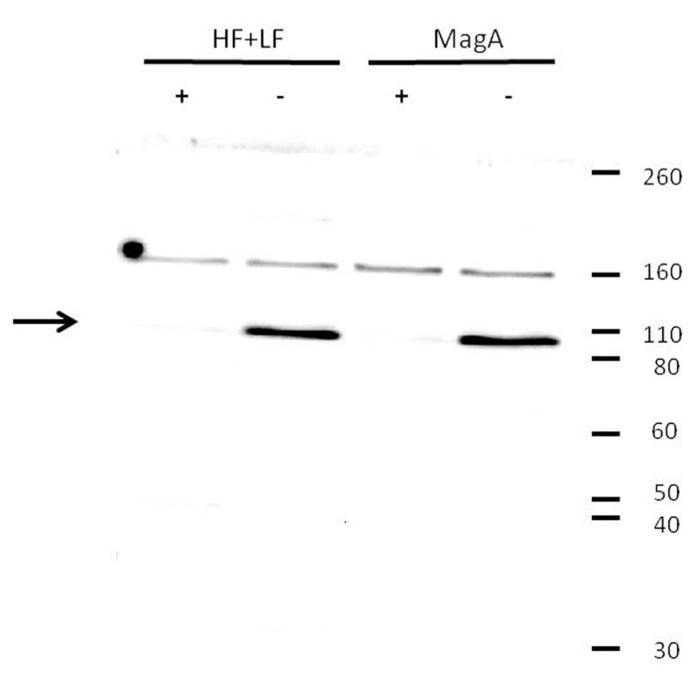
**Decrease in transferrin receptor expression upon iron supplementation.** Tumor cells were cultured in the presence (+) or absence (-) of iron supplementation, lysed, and analyzed by Western blot using a primary antibody to transferrin receptor. Both MagA- and HF + LF-expressing cells showed greater immunoreactivity toward the soluble form of transferrin receptor (arrow) in cells cultured in the absence of iron supplementation. Under reducing conditions, soluble transferrin receptor migrates at a M.W. of approximately 95K. Protein M.W. standards are indicated on the right margin.

The influence of MagA and HF + LF expression systems on the level of transferrin receptor was evaluated by Western blot (**Figure 4**). Both MagA- and HF + LF-expressing cells exposed to long-term iron-supplemented culture exhibited decreases in transferrin receptor expression. A densitometric analysis of the soluble transferrin receptor band at 95K indicates comparable protein levels in non-supplemented cell culture and an approximately 10-fold decrease in transferrin receptor in the presence of iron supplementation (**Table 5**).

**Table 5:** Relative expression of transferrin receptor^a^.

Sample	Pixel Number^b^	Relative Expression^c^
MagA	71254	14
MagA + Fe	4938	1.0
HF + LF	72700	15
HF + LF + Fe	7432	1.5

a Comparable results were obtained in four separate experiments.

b Pixel number was assessed using GeneTools software.

c Each sample consisted of 50 μg total protein.

## DISCUSSION

Improvements in molecular MRI are likely to evolve as developments in MR hardware, sequences and contrast agents progress (Huang et al., 2012). We are interested in adapting select features of the bacterial magnetosome to generate magnetosome-like nanoparticles in mammalian cells and provide MR contrast enhancement that is subject to genetic control. Toward this goal, we have used the gene *MagA* to examine (1) the potential for contrast enhancement in tumor cells and (2) whether iron binding protein from magnetotactic bacteria is compatible with mammalian iron regulation.

### RELAXATION RATES, IRON STORES AND MR CONTRAST

Using phantoms of compact cell pellets, we report cellular MR contrast using relaxation rate mapping and compare overexpression of MagA to HF + LF, the modified human ferritin subunits that lack iron response elements to enable continuous expression. In agreement with the literature, we show that there is little, if any, influence of cellular iron on the longitudinal relaxation rate (**Table 1**; Bin Na et al., 2009). Rather, our results show that transverse relaxation rates detect significant contrast enhancement in both MagA- and HF + LF-expressing cells when cultured in iron-supplemented medium (**Figures 1** and **2**, **Tables 2** and **3**). In these cell types and culture conditions, the ratio of elemental iron/zinc increased over 20-fold (**Table 4**). In addition to establishing specific iron uptake, relatively constant levels of zinc are consistent with little or no redox cytotoxicity (Chung et al., 2006).

Iron-based contrast agents create hypointense regions and cells with this type of contrast may be difficult to differentiate from anatomical regions that are also dark on MRI, such as the liver or a blood clot (Kim et al., 2011). For this reason, MagA and HF + LF overexpression may be useful in tracking breast cancer metastasis in the white matter of brain (Renier et al., 2010). In addition, the clear response to an iron-supplement suggests that these methods of gene-based iron contrast may be suitable indicators of hemorrhage, revascularization or changes in iron homeostasis. For example, Shpyleva et al. (2011) have reported changes in the level of ferritin subunits between epithelial and mesenchymal breast tumor cell lines (Shpyleva et al., 2011). In this case, to avoid interfering with the cancer cell biology, MagA overexpression might be a preferred method of cell tracking.

R2 measurements have previously been reported in 293FT and A549 cells expressing MagA (approx. 20 s^-^^1^, 3 T, *n* = 4; Zurkiya et al., 2008) and HF + LF (approx. 45 s^-^^1^, 11.7 T, *n* = 3; Genove et al., 2005), respectively. The values for MagA-expressing cells compare well with our findings; however, we show that R2 alone provides the weakest index of MR contrast activity (**Table 2**). Measures of R2* and R2′ report the most statistically significant change in cellular contrast upon iron supplementation. In addition, relative changes in R2′ were larger than those in R2, suggesting the potential of R2′ for better iron-related specificity, as previously suggested for human brain regions with high iron (Gelman et al., 1999). This may be important for *in vivo* cell tracking and optimization of molecular MRI. Rohani et al. (2013) used balanced SSFP to measure an increase in MagA- and HF + LF-expressing tumor contrast in the first 3 weeks of xenograft growth. Beyond this timeframe, contrast in the parental tumor increased, reducing the benefit of contrast gene expression in larger tumors. Relaxation mapping using optimal measures provides an additional tool for improving image analysis. As well, the iron-related contribution to transverse relaxation increases with field strength, thus, differences between contrast gene expression and controls should be greater at higher fields. We previously reported contrast enhancement in iron-supplemented, MagA-expressing N2A cells using 11T micro-MRI and a novel, dual echo sequence (Goldhawk et al., 2009).

### ATP STORES, CELLULAR VIABILITY, AND IRON HOMEOSTASIS

Although transverse relaxation rates vary as a function of tissue iron (Wood et al., 2005), R2 in particular is affected by other factors in the tissue or cell, such as water content reflecting proton density, subcellular compartmentalization, and water–protein interactions, particularly those that influence ferromagnetic metals like iron. By comparison, R2′ is mostly influenced by sources of magnetic field inhomogeneity. If sources of macroscopic inhomogeneity are minimized, as done here by using a spherical phantom, then R2′ should be influenced mostly by microscopic sources of inhomogeneity, which in our samples should be from iron particles. Post-processing methods have been developed to correct for macroscopic inhomogeneities, using the phase of the gradient echo signals for which no additional acquisition is required. This was previously demonstrated in a small animal imaging study reporting R2* and R2′ measurements from cancer cells labeled with superparamagnetic iron nanoparticles (Kuhlpeter et al., 2007). To further address variability in MR relaxation rates obtained from MagA and HF + LF overexpression, we examined ATP and transferrin receptor levels in cells cultured in the presence and absence of iron supplementation.

ATP quantification showed no significant difference in the energy stores of parental, MagA- and HF + LF-expressing cells, regardless of iron supplementation (**Figure 3**). Iron uptake in both prokaryotes and mammalian cells is an ATP-dependent process. In bacteria, iron transport requires ATP hydrolysis (Andrews et al., 2003). When MagA was expressed in *E. coli*, iron uptake was observed in membrane vesicles when cells were supplemented with iron and ATP; however, this response was limited when ATP was excluded, suggesting that MagA function is coupled to ATP hydrolysis (Nakamura et al., 1995a). In mammalian cells, transferrin-bound iron is transported into the cell through receptor-mediated endocytosis. Iron is released from the transferrin-transferrin receptor complex by a pH change in the endosomal compartment, caused by proton-pump ATPase activity (Anderson and Vulpe, 2009). The results presented in the current report confirm that iron loading in our engineered tumor cell model produced neither cytotoxicity nor substantial changes in total ATP stores.

Transferrin receptor, the principle mammalian iron import mechanism, is ubiquitously expressed in almost all cells (Ponka and Lok, 1999), with higher levels of expression in highly proliferating cells and those that have a functional need for iron (Pantopoulos et al., 2012). Compared to healthy tissue, tumor cells have reduced levels of ferritin expression and elevated levels of transferrin receptor (Daniels et al., 2006; Anderson et al., 2012). Such changes in the regulation of iron uptake, storage and distribution support rapid proliferation, for example by supplying iron as a cofactor for ribonucleotide reductase and DNA synthesis (Weinberg, 1992; Nyholm et al., 1993). The tendency of proliferating cells toward iron uptake may be used to best advantage in cancer cell tracking by MRI and the development of gene-based contrast. In MagA- and HF + LF-expressing tumor cells, we have detected a decrease in transferrin receptor following culture in the presence of iron supplementation (**Figure 4**, **Table 5**). Therefore, the activity of MagA has elicited the same homeostatic response as expected of an increase in ferritin storage. In spite of this, a statistically significant level of MR contrast enhancement was achieved with both expression systems, verifying dysregulation of HF + LF from iron response elements and suggesting that MagA may function outside the regulatory control of iron binding proteins. In the case of MagA and HF + LF activity, the lack of cellular regulation may be reflected in greater fluctuation in iron levels and therefore in MR contrast. The iron export activity of ferroportin (Ganz, 2005) in MagA- and HF + LF-expressing cells has not been reported.

The ability of MagA to circumvent key features of mammalian iron regulation, without causing cytotoxicity, bodes well for future development of gene-based contrast. Effective MR contrast in mammalian cells, derived from the overexpression of iron handling protein(s), would benefit from a clearer understanding of which combination of magnetotactic bacterial or magnetosome genes will (1) sequester iron within a membrane-enclosed vesicle and (2) permit iron biomineralization in the presence of mammalian iron homeostasis. What is the simplest magnetosome unit? The definition awaits further insight into the synthesis of this compartment and may vary depending on the application. Magnetosome gene knock-out studies (Greene and Komeili, 2012) raise the possibility that a subset of genes may be used to generate a minimal compartment in multiple cell types that would permit iron biomineralization for non-invasive medical imaging with MRI.

## AUTHOR CONTRIBUTIONS

Both Anindita Sengupta and Karina Quiaoit are jointly supervised by Neil Gelman and Donna E. Goldhawk and contributed to the reporting of this data. Relaxation rate mapping and ATP assays were performed by Anindita Sengupta and Karina Quiaoit, respectively. Neil Gelman, R. Terry Thompson and Frank S. Prato provided scientific input. Donna E. Goldhawk wrote the manuscript and coordinated the analysis of cellular iron and transferrin receptor.

## Conflict of Interest Statement

Donna E. Goldhawk, R. Terry Thompson and Frank S. Prato are authors on a patent filing describing the use of magnetosome genes in eukaryotic cells (European Patent # 2029754).
